# Immunoglobulin A nephropathy in a patient with neurofibromatosis type 1: A case report and literature review

**DOI:** 10.1097/MD.0000000000027572

**Published:** 2021-10-22

**Authors:** Harin Rhee, Sungmi Kim, Wanhee Lee, Hakeong Jeon, Da Woon Kim, Byung-Min Ye, Hyo Jin Kim, Min Jeong Kim, Seo Rin Kim, Il Young Kim, Sang Heon Song, Eun Young Seong, Dong Won Lee, Soo Bong Lee

**Affiliations:** a Department of Internal Medicine, Pusan National University School of Medicine, Yangsan, Republic of Korea; b Division of Nephrology, Biomedical Research Institute, Pusan National University Hospital, Pusan, Republic of Korea; c Research Institute for Convergence of Biomedical Science and Technology, Pusan National University Yangsan Hospital, Yangsan, Republic of Korea.

**Keywords:** IgA nephropathy, IgAN, immunoglobulin A nephropathy, neurofibromatosis type 1, NF-1

## Abstract

**Rationale:**

Neurofibromatosis type 1 (NF-1) is an autosomal-dominant neurocutaneous disorder that affects the skin, bones, and nervous system. The most common manifestation of kidney involvement is renal artery stenosis; glomerulonephritis is extremely rare. In this case report, we present a patient with NF-1 and immunoglobulin A nephropathy (IgAN).

**Patient concerns:**

A 51-year-old Korean man previously diagnosed with NF-1 presented with persistent proteinuria and hematuria identified during a routine medical check-up. He had no history of hypertension or diabetes, and denied a history of alcohol use or smoking.

**Diagnosis:**

The contrast-enhanced computed tomography scan revealed normal-sized kidneys and no evidence of renal artery stenosis. On the day of the kidney biopsy, laboratory tests showed a serum creatinine level of 1.1 mg/dL, urine protein/creatinine ratio of 1.3 g/g, and urine red blood cell count of >10 to 15/HPF. The kidney biopsy sample revealed IgAN grade III, according to Lee glomerular grading system.

**Intervention:**

The patient was advised to take 4 mg of perindopril.

**Outcome:**

Three months after the treatment, the urine protein/creatinine ratio decreased to 0.6 g/g, with no change in the serum creatinine level (1.03 mg/dL).

**Lessons:**

A genetic link between NF-1 and IgAN or other glomerular diseases is not established. However, activation of the mTOR pathway may explain this association.

## 1 Introduction

Neurofibromatosis type 1 (NF-1) is a rare genetic disorder that affects 1 out of every 3000 live births.^[1]^ NF-1 is an autosomal-dominant neurocutaneous disorder that affects the skin, bones, and nervous system.^[1,2]^ Although kidney involvement is rare, it most commonly presents as renal artery stenosis,^[3,4]^ which is reported in less than 1% of cases.^[3]^ Glomerulonephritis is rare in NF-1 patients; only a few cases of membranous nephropathy^[5–7]^ and focal segmental glomerulosclerosis,^[8–10]^ and 3 of immunoglobulin A nephropathy (IgAN),^[11,12]^ have been reported in NF-1 patients. In this case report, we present the case of a 51-year-old Korean man with NF-1 and IgAN.

## 2 Case presentation

A 51-year-old Korean man presented to the nephrology outpatient clinic with proteinuria and hematuria for more than 9 years. Nine years ago, the patient was diagnosed with NF-1 based on the presence of several café au lait macules and cutaneous neurofibromas. A next-generation sequencing panel test confirmed a NF-1 gene mutation on chromosome 17. At the time of diagnosis, proteinuria (urine protein/creatinine ratio = 0.3 g/g; reference value < 0.15) and hematuria (red blood cell [RBC] count = 10–15/HPF; reference value < 0–2) were present. However, the patient was not evaluated by a nephrologist because he had a normal serum creatinine level (0.64 mg/dL; reference value = 0.4–1.2). He had 1 daughter diagnosed with NF-1 during infancy. None of his ancestors were diagnosed with NF-1.

The patient's automated blood pressure in the seated position was 134/99 mm Hg and the pulse rate was 71 beats/min. He denied a history of smoking or alcohol consumption. His laboratory tests showed a serum creatinine level of 1.21 (reference value = 0.4–1.2) mg/dL, urine protein/creatinine ratio of 1.3 (reference value < 0.15) g/g, and urine RBC count > 100/HPF (reference value < 0–2). A kidney biopsy was planned due to suspicion of glomerulonephritis.

Three days later, the patient was admitted to the hospital. At admission, his blood pressure was 110/70 mm Hg in the supine position, the heart rate was 74 beats per minute, and the body temperature was 36.2°C. Physical examination revealed café au lait spots and multiple neurofibromas on the abdomen, back, and arms. Serologic tests were negative for anti-neutrophilic cytoplasmic antibody, antinuclear antibody, anti-phospholipid 2 receptor antibody, anti-glomerular basement membrane antibody, rheumatoid factor, serum/urine protein electrophoresis, and hepatitis B and C. Complement 3 and 4 levels were normal. His biochemical tests showed a hemoglobin level of 12.4 (reference value = 13.5–17.5) g/dL, platelet count of 230 × 10E^3^/μL (reference value = 140–420), albumin level of 4.1 (reference value = 3.3–5.2) g/dL, creatinine level of 1.1 (reference value = 0.4–1.2) mg/dL, cystatin C level of 1.28 (reference value = 0.61–0.95) mg/dL, CysC-Cr-based eGFR (as determined by the CKD-EPI equation) of 66.3 (reference value > 60) mL/min/1.73 m^2^, sodium level of 134.5 (reference value = 138–148) mmol/L, and potassium level of 4.52 (reference value = 3.5–5.3) mmol/L. Urinalysis showed a protein/creatinine ratio of 0.9 (reference value < 0.15) g/g and an RBC count of 15 to 20/HPF (reference value < 0–2). Contrast-enhanced abdominal computed tomography showed no evidence of renal artery stenosis. In the reconstructed view, the right and left kidneys were estimated to be 10.5 and 11.2 cm long, respectively.

**Figure 1:**
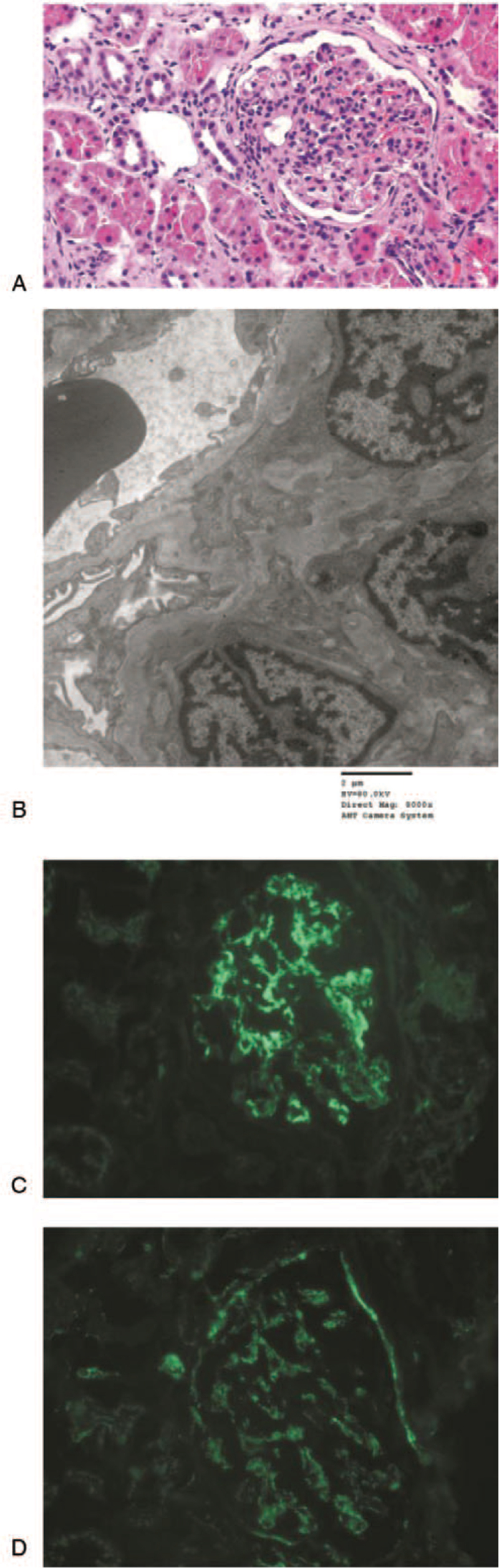
Hematoxylin and eosin-stained light microscopy image showing mesangial hypercellularity (magnification: ×400) (A); Electron microscopy image showing localized mesangial deposits (magnification: ×8000) (B); Immunofluorescent microscopy image showing +3 diffuse mesangial staining for immunoglobulin A (C); and +1 diffuse mesangial staining for C3 (D).

A kidney biopsy was performed and 3 cores of kidney tissue were obtained. Each core was evaluated by light and electron microscopy and immunofluorescence analysis. On light microscopy, 20 glomeruli were observed and 5 (25%) of them showed global sclerosis. The glomeruli were slightly increased in size and moderately hypercellular, with an increase in mesangial cells and the mesangial matrix also seen (Fig. 1A). Four glomeruli (20%) exhibited segmental sclerosis. Tubules had moderate to severe focal atrophy and loss, with interstitial infiltration of mononuclear cells and fibrosis. Mild arteriolosclerosis was also noted. On electron microscopy, localized tiny mesangial deposits were observed (Fig. 1B). The glomerular basement membrane exhibited normal thickness with widening due to severe focal subendothelial edema. Epithelial cell foot processes showed focal marked effacement. The sections for immunofluorescent microscopy contained 3 glomeruli. The mesangium diffusely stained for immunoglobulin A (+3; IgA) (Fig. 1C), C3 (+1) (Fig. 1D), and lambda (+2–3). Tests for IgG, IgM, C1q, C4, fibrinogen, and kappa were negative. The aforementioned findings were consistent with a diagnosis of IgAN grade III, according to Lee glomerular grading system. At discharge, the patient was prescribed 4 mg/day of perindopril.

Three months after hospital discharge, the patient was followed up in the outpatient clinic. The urine protein/creatinine ratio decreased to 0.6 (reference value < 0.15) g/g, and the serum creatinine level remained stable at 1.03 (reference value = 0.4–1.2) mg/dL. The patient was advised to attend long-term follow-up visits to monitor his renal function.

## 3 Discussion and conclusions

The first cases of IgAN in NF-1 patients were reported in 1997 from Japan, in a 41-year-old female and her 24-year-old son.^[11]^ The mother presented with proteinuria and occult hematuria for 1 year before the diagnosis of NF-1. Renal biopsy revealed moderate IgAN. Her renal function gradually deteriorated, and repeat biopsy revealed marked sclerosis of the glomeruli, tubules, and interstitium. Her son underwent a kidney biopsy for proteinuria and occult hematuria 5 years after the NF-1 diagnosis. At that time, his serum creatinine was 1.25 mg/dL, and he had proteinuria (3.3 g/d). The kidney biopsy specimen confirmed a diagnosis of IgAN with fibrocellular crescents. The authors of this report did not suggest a genetic relationship between IgAN and NF-1.

Another case of IgAN was reported in 2020,^[12]^ in a 56-year-old Japanese woman with NF-1. She presented with new-onset microscopic hematuria and arthralgia. She also had palpable purpura on the lower parts of her legs. Her kidney and skin biopsy samples revealed IgA vasculitis. A genetic relationship between NF-1 and IgA vasculitis was not suggested by the authors.

In our patient, IgAN was diagnosed 9 years after genetic confirmation of NF-1. However, hematuria and proteinuria had been present since the diagnosis of NF-1, so IgAN may have been present before the pathologic confirmation. We excluded all possible causes of secondary IgAN,^[13]^ including liver cirrhosis, inflammatory bowel disease, hepatitis B and C, autoimmune diseases, malignancies, psoriasis, sarcoidosis, cystic fibrosis, and pulmonary fibrosis. At the time of kidney biopsy, his serum creatinine level was 1.1 mg/dL and the urine protein/creatinine ratio was 0.9 g/g; this had worsened over the previous 9 years.

Among the 4 reported cases of IgAN with NF-1, 2 had progressive renal dysfunction, and 1 exhibited accelerated inflammation at the time of kidney biopsy. Compared to the natural course of IgAN in the general population,^[14]^ NF-1 patients seemed to have a worse clinical condition.

Although there is no conclusive evidence of a genetic link between IgAN and NF-1, we suspect that NF-1 affects the development and progression of IgAN. NF-1 is caused by a mutation in the *NF-1* gene, located on the long arm of chromosome 17.^[15]^*NF1* gene encodes neurofibromin, which negatively regulates the Ras/PI3K signaling pathway that in turn regulates the mTOR pathway.^[16–19]^ Therefore, alteration of neurofibromin in NF-1 patients can cause aberrant activation of the mTOR pathway.^[16–19]^

Activation of the mTOR pathway has been reported in an experimental model of IgAN. In the experiment by Nagai et al,^[20]^ mesangial cell mTOR 1 activation induced mesangium expansion and increased the production of collagen IV, collagen I, and smooth muscle actin in glomeruli. Additionally, treatment with rapamycin, an mTOR inhibitor, suppressed mesangial expansion. In another experiment with an IgAN rat model, rapamycin attenuated IgA deposition in glomeruli and preserved the renal function.^[21]^

The multihit hypothesis is the most widely accepted with respect to IgAN pathogenesis.^[13,22]^ This framework involves aberrant glycosylation of IgA1, synthesis of antiglycan autoantibodies that target galactose-deficient IgA1 (Gd-IgA1), formation of circulating Gd-IgA1-antiglycan IgG immune complexes, and accumulation of nephritogenic complexes in the mesangium, which activate the inflammatory response that contributes to mesangial matrix production and mesangial cell proliferation. Even though the genetic link between IgAN and NF-1 is not established, we speculate that the inflammatory response to nephritogenic Gd-IgA1-antiglycan IgG immune complexes in mesangium is enhanced in NF-1 patients due to aberrant mTOR activation. Glomerulonephritis other than IgAN have also been reported in NF-1 patients, including membranous nephropathy,^[5,7,23]^ focal segmental glomerulosclerosis,^[8–10]^ immunoglobulin M nephropathy,^[24]^ C1q nephropathy,^[25]^ minimal change disease,^[26]^ and renal amyloidosis^[26]^ (Table 1). Activation of the mTOR pathway in NF-1 patients may increase susceptibility to diverse nephritogenic antigens.

**Table 1:** Reported cases of glomerular disease in patient with neurofibromatosis type 1.

Reference	Year	Country	Race	Sex	Age	Type of GN
Kokubo et al^[5]^	1993	Japan	Asian	F	68	MN
Toth et al^[7]^	1996	Hungary	Unknown	NA	NA	MN
Taniguchi et al^[11]^	1997	Japan	Asian	F	41	IgAN
Taniguchi et al^[11]^	1997	Japan	Asian	M	24	IgAN
Wani et al^[23]^	2006	India	Unknown	F	70	MN
Gersch et al^[8]^	2006	USA	White	M	22	FSGS
Tarrass^[9]^	2008	Morocco	Unknown	M	58	FSGS
Afshinnia et al^[10]^	2013	USA	White	F	42	FSGS
Chang et al^[24]^	2015	S. Korea	Asian	F	44	IgM nephropathy
Varyani et al^[25]^	2019	India	Unknown	M	51	C1q nephropathy
Orera et al^[26]^	2019	Spain	Unknown	F	41	MCD
Orera et al^[26]^	2019	Spain	Unknown	F	71	Renal amyloidosis
Shimamura et al^[12]^	2020	Japan	Asian	F	56	IgAN
Rhee et al	2021	S. Korea	Asian	M	51	IgAN

In this article, we report the case of a patient with NF-1 and IgAN. It is unclear whether the co-occurrence of NF-1 and IgAN was due to a genetic association or coincidence. However, activation of the mTOR pathway may reflect a possible genetic association. Further experimental and clinical studies are required to evaluate the potential genetic association between NF-1 and IgAN.

## Author contributions

**Conceptualization:** Harin Rhee.

**Investigation:** Sungmi Kim, Wanhee Lee, Hakeong Jeon.

**Validation:** Da Woon Kim, Byung-Min Ye, Hyo Jin Kim, Min Jeong Kim, Seo Rin Kim, Il Young Kim, Sang Heon Song, Eun Young Seong, Dong Won Lee, Soo Bong Lee.

**Writing – original draft:** Harin Rhee.

**Writing – review & editing:** Harin Rhee.

## References

[1] Karaconji T Whist E Jamieson RV Flaherty MP Grigg JRB. Neurofibromatosis type 1: review and update on emerging therapies. Asia Pac J Ophthalmol (Phila) 2019;8:62–72. 30387339 10.22608/APO.2018182

[2] Kim MJ Cheon CK. Neurofibromatosis type 1: a single center's experience in Korea. Korean J Pediatr 2014;57:410–5. 25324867 10.3345/kjp.2014.57.9.410 PMC4198956

[3] Han M Criado E. Renal artery stenosis and aneurysms associated with neurofibromatosis. J Vasc Surg 2005;41:539–43. 15838492 10.1016/j.jvs.2004.12.021

[4] Malav IC Kothari SS. Renal artery stenosis due to neurofibromatosis. Ann Pediatr Cardiol 2009;2:167–9. 20808634 10.4103/0974-2069.58323 PMC2922669

[5] Kokubo T Hiki Y Horii A Kobayashi Y. Recklinghausen's neurofibromatosis associated with membranous nephropathy. Nephron 1993;65:486 doi:10.1159/000187543. 8290010 10.1159/000187543

[6] Kokubo T Hiki Y Horii A Kobayashi Y. A case of Recklinghausen neurofibromatosis associated with membranous nephropathy. Nihon Jinzo Gakkai Shi 1994;36:951–4. 7933673

[7] Toth T Trinn C Simon L Nagy J. A case of membranous glomerulonephritis associated with Recklinghausen's neurofibromatosis. Clin Nephrol 1996;45:271–2. 9156954

[8] Gersch MS Talor Z. Focal segmental glomerular sclerosis in a patient with neurofibromatosis type I. Am J Kidney Dis 2006;47:e17–9. 16377376 10.1053/j.ajkd.2005.09.017

[9] Tarrass F. Focal and segmental glomerulosclerosis and von Recklinghausen's neurofibromatosis: coincidental or associated? Saudi J Kidney Dis Transpl 2008;19:453–4. 18445911

[10] Afshinnia F Vega-Warner V Killen P. Focal segmental glomerulosclerosis in association with neurofibromatosis type 1: a case report and proposed molecular pathways. Clin Kidney J 2013;6:208–10. 23805377 10.1093/ckj/sft010 PMC3693487

[11] Taniguchi Y Yorioka N Kanbe M. Parent and child cases of IgA nephropathy associated with von Recklinghausen's disease. Nephron 1997;75:113–4. 9031286 10.1159/000189515

[12] Shimamura Y Ogawa Y Shinohara T. Immunoglobulin A vasculitis in a patient with neurofibromatosis type 1. Nefrologia (Engl Ed) 2020;40:676–7. 32067820 10.1016/j.nefro.2019.10.008

[13] Pattrapornpisut P Avila-Casado C Reich HN. IgA nephropathy: Core Curriculum 2021. Am J Kidney Dis 2021;78:429–41. 34247883 10.1053/j.ajkd.2021.01.024

[14] D’Amico G. Natural history of idiopathic IgA nephropathy and factors predictive of disease outcome. Semin Nephrol 2004;24:179–96. 15156525 10.1016/j.semnephrol.2004.01.001

[15] Viskochil D Buchberg AM Xu G. Deletions and a translocation interrupt a cloned gene at the neurofibromatosis type 1 locus. Cell 1990;62:187–92. 1694727 10.1016/0092-8674(90)90252-a

[16] Bergoug M Doudeau M Godin F Mosrin C Vallee B Benedetti H. Neurofibromin structure, functions and regulation. Cells 2020;9:2365 doi:10.3390/cells9112365. 10.3390/cells9112365 PMC7692384 33121128

[17] Martin GA Viskochil D Bollag G. The GAP-related domain of the neurofibromatosis type 1 gene product interacts with ras p21. Cell 1990;63:843–9. 2121370 10.1016/0092-8674(90)90150-d

[18] Xu GF O’Connell P Viskochil D. The neurofibromatosis type 1 gene encodes a protein related to GAP. Cell 1990;62:599–608. 2116237 10.1016/0092-8674(90)90024-9

[19] Bollag G McCormick F. Ras regulation. NF is enough of GAP. Nature 1992;356:663–4. 1570011 10.1038/356663a0

[20] Nagai K Tominaga T Ueda S. Mesangial cell mammalian target of rapamycin complex 1 activation results in mesangial expansion. J Am Soc Nephrol 2017;28:2879–85. 28701517 10.1681/ASN.2016111196 PMC5619961

[21] Tian J Wang Y Liu X Zhou X Li R. Rapamycin ameliorates IgA nephropathy via cell cycle-dependent mechanisms. Exp Biol Med (Maywood) 2015;240:936–45. 25349217 10.1177/1535370214555666 PMC4935411

[22] Suzuki H Kiryluk K Novak J. The pathophysiology of IgA nephropathy. J Am Soc Nephrol 2011;22:1795–803. 21949093 10.1681/ASN.2011050464 PMC3892742

[23] Wani MM Reshi AR Banday KA Najar MS. von Recklinghausen's neurofibromatosis associated with membranous glomerulonephritis. Saudi Med J 2006;27:534–5. 16598334

[24] Chang KY Kim HW Kim YS. Case of immunoglobulin M nephropathy in a patient with neurofibromatosis type 1. Nephrology (Carlton) 2015;20:666–7. 26278121 10.1111/nep.12487

[25] Varyani UT Shah NM Shah PR Kute VB Balwani MR Trivedi HL. C1q nephropathy in a patient of neurofibromatosis type 1: a rare case report. Indian J Nephrol 2019;29:125–7. 30983754 10.4103/ijn.IJN_353_17 PMC6440327

[26] Orera A Lacarra S Fernandez L Gomez N Slon MF Manrique J. Secondary glomerulonephritis in neurofibromatosis type 1. Two case reports. An Sist Sanit Navar 2019;42:345–9. 31859277 10.23938/ASSN.0720

